# Cytoreductive surgery and hyperthermic intraperitoneal chemotherapy for pseudomyxoma peritonei in a liver-transplanted patient: a case report

**DOI:** 10.1186/s12957-018-1482-7

**Published:** 2018-09-05

**Authors:** Ebbe Billmann Thorgersen, Espen Melum, Trine Folseraas, Stein Gunnar Larsen, Pål Dag Line

**Affiliations:** 1 Department of Gastroenterological Surgery, Division of Surgery, Inflammatory Diseases and Transplantation, The Norwegian Radium Hospital Oslo University Hospital, Pb. 4950 Nydalen, N-0424 Oslo, Norway; 2 Institute of Immunology, Oslo University Hospital Rikshospitalet and University of Oslo, Oslo, Norway; 3 Norwegian Primary Sclerosing Cholangitis Research Center, Department of Transplantation Medicine, Division of Surgery, Inflammatory Diseases and Transplantation, Oslo University Hospital Rikshospitalet, Oslo, Norway; 4 Research Institute of Internal Medicine, Division of Surgery, Inflammatory Diseases and Transplantation, Oslo University Hospital, Oslo, Norway; 5 Institute of Clinical Medicine, University of Oslo, Oslo, Norway; 6 Section for Gastroenterology, Department of Transplantation Medicine, Division of Surgery, Inflammatory Diseases and Transplantation, Oslo University Hospital Rikshospitalet, Oslo, Norway; 7 Section for Transplantation Surgery, Department of Transplantation Medicine, Oslo University Hospital, Oslo, Norway

**Keywords:** Cytoreductive surgery, Hyperthermic intraperitoneal chemotherapy, Liver transplantation, Primary sclerosing cholangitis, Pseudomyxoma peritonei

## Abstract

**Background:**

Diagnostic work-ups in transplanted immunosuppressed patients are a challenge as non-specific findings may be interpreted as transplant-related complications. If the disease in question is rare and slowly developing like pseudomyxoma peritonei (PMP), it is even more difficult. Cytoreductive surgery (CRS) and subsequent hyperthermic intraperitoneal chemotherapy (HIPEC) is the recommended treatment for PMP even with extensive peritoneal spread. CRS-HIPEC for PMP after liver transplantation (LTX) has not been described before.

**Case presentation:**

A 48-year-old female patient with end-stage primary sclerosing cholangitis (PSC) underwent orthotopic LTX and subsequent pancreaticoduodenectomy after the finding of cholangiocarcinoma in situ in the native common bile duct. Ten years after the transplantation, she developed symptoms and signs suspected to represent graft-related complications. An extensive work-up revealed PMP. Upon reassessment, a cystic mass near the coecum could be seen on computed tomography scan 1 year after transplantation. The multidisiplinary team was hesitant to accept the patient for CRS-HIPEC because of extensive PMP and possible risk to the graft. However, she was eventually accepted and underwent the procedure. The Peritoneal Cancer Index (PCI) was 28 of 39, and surgical debulking was performed followed by HIPEC. The transplant control 2 months after surgery showed no harm to the graft.

**Conclusions:**

Previous LTX should not exclude the possibility for CRS-HIPEC in PMP, even with extensive burden of disease.

## Background

Primary sclerosing cholangitis (PSC) is an inflammatory disease of the bile ducts leading to concentric fibrosis [1]. The etiology is unknown, but an autoimmune component likely contributes [2]. No medical treatment slow disease progression to liver failure; hence, PSC is an important indication for liver transplantation (LTX), not least in Northern Europe [3]. PSC predispose to hepatobiliary and colorectal cancers [4].

Pseudomyxoma peritonei (PMP) is a rare, slowly progressive mucinous malignant disease confined to the peritoneal cavity, usually originating from the appendix [5]. The predisposed areas within the peritoneal cavity of PMP are defined by fluid absorption through lymphatic lacunae and lymphoid aggregates (greater omentum, right hemidiaphragm) and gravity (pelvic cavity, paracolic gutters) [6]. The natural course of the disease is slow progression of peritoneal mucinous masses until death caused by complications due to the bulk of tumor.

## Case presentation

**Fig. 1:**
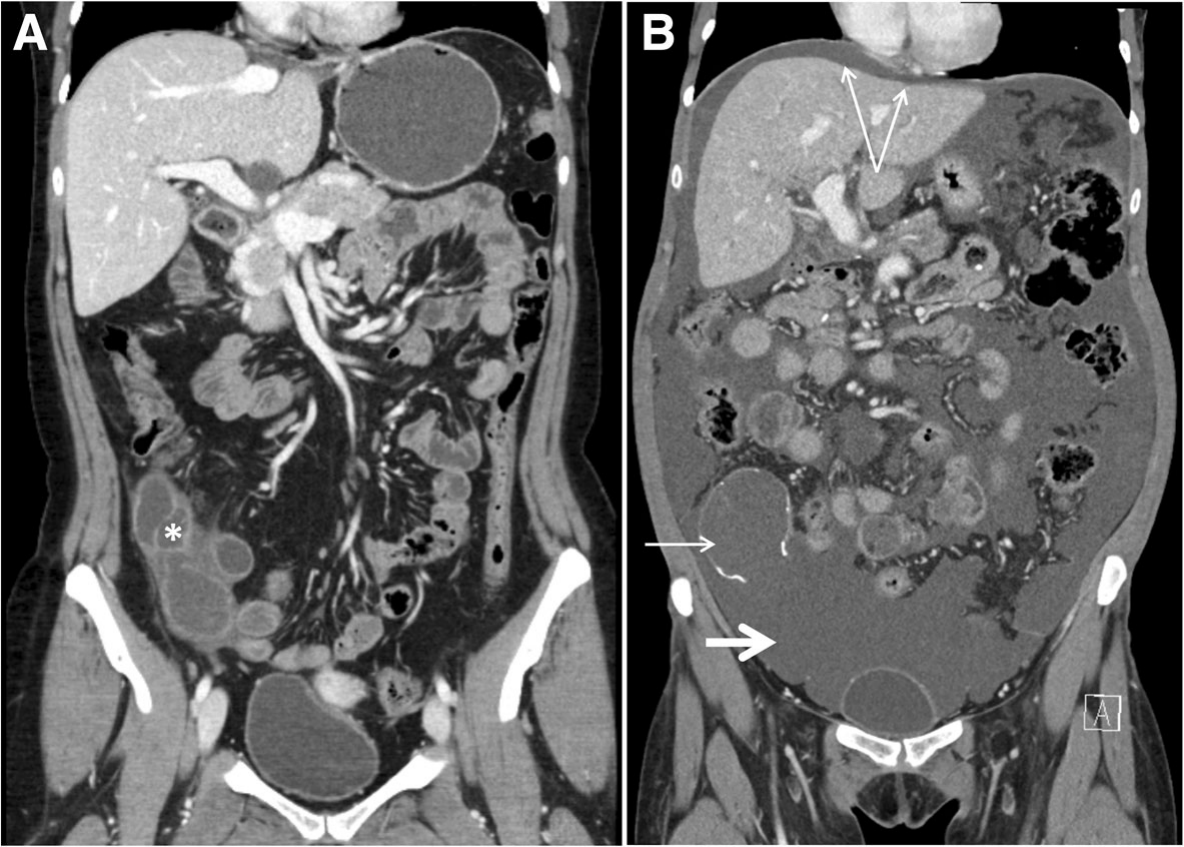
Development of pseudomyxoma peritonei (PMP). CT scans with coronal view before (**a**) and after (**b**) development of PMP. Mucinous cystadenoma of the appendix (asterisk) in **a**. Typical features of PMP (**b**): ruptured calcified appendix (short slim arrow), massive mucinous ascites (short thick arrow), central displacement of the small bowel, and visceral scalloping of the liver (double arrow)

A 48-year-old female patient underwent her first work-up for possible liver disease 30 years ago. Based on findings of chronic active hepatitis in liver biopsy, autoimmune hepatitis was suspected, and treatment with prednisolone and azathioprine initiated. Seven years later, an endoscopic retrograde cholangiopancreatography (ERCP) showed a stricture in the common bile duct which was interpreted as possible extrahepatic PSC or tentatively an inborn anomaly of the common bile duct. After 20 years with fluctuating transaminases between 100 and 300 U/L and no recorded major clinical events, she developed general edema and thoracic spider nevi. A computed tomography (CT) scan revealed liver cirrhosis and portal hypertension with massive ascites. After an initial response to symptomatic treatment, she soon developed uncompensated liver failure with severe hepatic encephalopathy and was transferred to the national transplant unit for work-up and listing for liver transplantation. A few days later, an ABO-identical donor liver was available and LTX was performed with a duct-to-duct biliary anastomosis. The histopathological examination of the liver explant revealed cirrhosis and pathological changes typical for underlying PSC. Cholangiocarcinoma in situ was found in the common hepatic duct, the cystic duct, and the common bile duct, involving the resection margin. It was decided that the patient should be controlled with ERC with brush cytology from the duct-to-duct anastomosis after 6 months due to the histopathological findings. The brush cytology showed mild to moderate epithelial dysplasia in the common bile duct. After discussion in the multidisciplinary team, a pancreaticoduodenectomy (Whipple procedure) was decided due to the risk of development of cholangiocarcinoma. Ten months after LTX, the procedure was performed. Because of a possible increased risk of anastomotic leak due to immunosuppression, an enteropancreatic anastomosis was avoided and the pancreatic duct was occluded. The surgical specimen showed intestinal metaplasia in the common bile duct, but no dysplasia or carcinoma. She was then followed according to standard post-liver transplantation follow-up regimen. The patient developed insulin-dependent diabetes mellitus and received tuberculostatic treatment due to disseminated tuberculosis (pulmonary and meningeal), but the graft function remained good. At the 5-year post-transplantation control, recurrent PSC in the graft was diagnosed on liver biopsy. In addition, a 10 × 4 cm multicystic mass in the lower right abdominal quadrant was described on the routine abdominal ultrasound (US) exam. Upon reassessment, a CT scan of the abdomen performed before the Whipple procedure also showed a 10 × 5 cm multicystic mass in the same region. The lesion was asymptomatic, and based on imaging, the lesion was considered to be a benign gynecological cyst. Consequently, no further investigations were performed to establish the exact etiology. At 10 years of follow-up, the patient reported increasing abdominal distension, anorexia, weight loss, and fatigue for the past 6 to 8 months. Clinical examination revealed extensive sarcopenia, multiple spider nevi, and tense ascites. Biochemical tests showed an iron deficiency anemia with Hgb of 9.0 g/dL, moderately elevated liver function tests, inflammatory markers, and carcinoembryonic antigen (CEA). Imaging, including liver and abdominal US, abdominal and thoracic CT, CT enterography, and positron emission tomography (PET)-CT unveiled liver graft fibrosis/cirrhosis with features of portal hypertension, massive low-attenuating heterogeneous ascites, a focal wall thickening in the middle part of the jejunum, multiple focal lesions in both lungs, a focal lesion in the thyroid gland, and the previously described cystic mass in the lower right abdominal quadrant. Diagnostic abdominal paracentesis revealed thick gelatinous ascites, which was culture negative. The ascites was rich in mucin, but without neoplastic cells on repeated cytology examinations. The provisional primary diagnosis of graft fibrosis-related portal hypertension with ascites could not be supported. Further extensive diagnostic work-up did not confirm tentative diagnoses such as gastrointestinal tuberculosis, sarcoidosis, mucinous tumors of the ovaries, or other causes of peritoneal carcinomatosis. Bearing in mind the differential diagnosis of PMP as a rare, but relevant tentative cause of mucinous ascites, re-evaluation of the contrast-enhanced CT scan raised the suspicion of PMP and that the cystic lesion actually represented a mucinous cystadenoma of the appendix (Fig. 1).

**Fig. 2:**
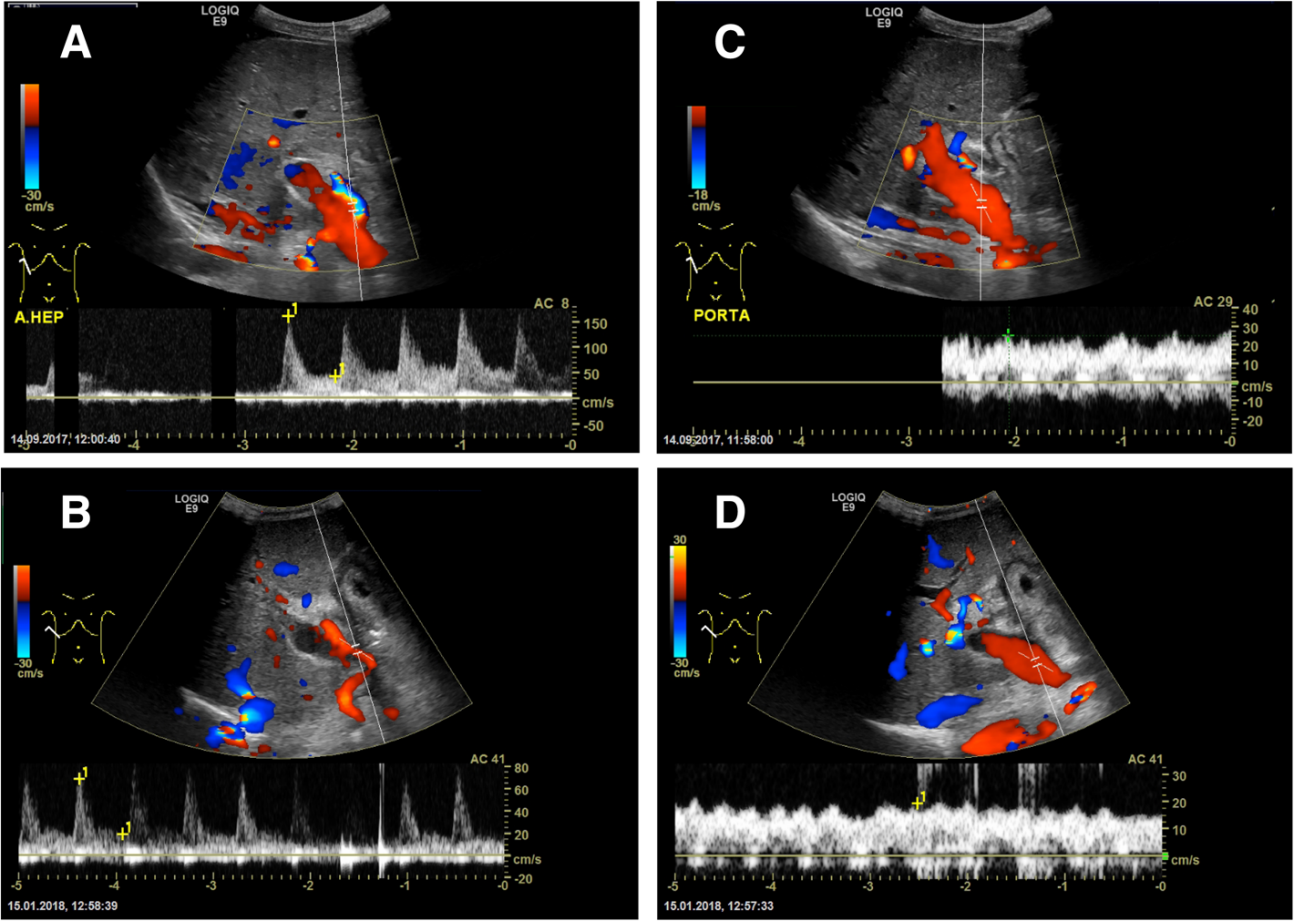
Circulation of the liver graft. The pictures to the left show examination of the common hepatic artery of the liver graft with US Doppler before (**a**) and after (**b**) CRS-HIPEC with a resistive index (RI) of 0.73 and 0.72 respectively. The pictures to the right show US Doppler examination of the portal vein of the liver graft before (**c**) and after (**d**) CRS-HIPEC with no resistance found in either exam

The multidisciplinary team initially turned down cytoreductive surgery and hyperthermic intraperitoneal chemotherapy (CRS-HIPEC) for the patient due to concerns of the magnitude of the disease with the risk of harming the graft during the procedure. However, after a renewed assessment, the patient was accepted for explorative laparotomy and potentially CRS-HIPEC. Perioperatively, the peritoneal cancer index (PCI) was estimated to 28 of 39 and it was found that CRS-HIPEC was technically doable and meaningful. The operation time was approximately 11 h. Surgery included removal of considerable amounts of mucinous masses, extensive peritonectomy including the right hemidiaphragm, part of the left hemidiaphragm and the pelvis, omentectomy, hysterectomy, bilateral salpingo-oophorectomy, ileocaecal resection, splenectomy, and removal of the umbilicus. Minimal mucinous masses in relation to the piggyback anastomosis to the inferior caval vein were left due to safety reasons. HIPEC was then performed with mitomycin C for 90 min. The histopathological examination showed perforated low-grade appendiceal mucinous neoplasm and wide-spread mucin without viable tumor cells in all abdominal specimens, compatible with PMP. Forty-two days after surgery, the patient was readmitted with urinary tract infection, anemia, high glucose levels, reversible kidney failure, weight loss, and malnutrition. She recovered on antibiotics and supportive treatment and received follow-up by nutritionists after discharge. Two months after surgery, she had started regaining weight and transplant control displayed satisfactory graft circulation and function (Fig. 2).

## Discussion

The diagnosis of PMP can be a challenge as is clearly demonstrated in the present case history. PMP is rare with an estimated incidence of 2–4 per million per year [7, 8]. The natural history of the disease is of slow progression, and the patients may not have symptoms for many years. The eventual symptoms are often vague, if the debut is not acute appendicitis. Common presentations are abdominal distension, new onset hernia, and diffuse abdominal pain [9]. In this case, the previous LTX made the work-up difficult, as most of the symptoms the patient displayed including ascites could be graft-related complications. It was also clear that PSC had relapsed in the graft as demonstrated by previous histology and graft-failure was therefore the initial first suspicion.

A CT scan is the recommended radiological modality for PMP [10]. In the presented patient, several other radiological examinations, like US Doppler and magnetic resonance imaging (MRI) of the liver graft and bile ducts and PET-scan were conducted before a proper abdominal contrast-enhanced CT scan was done and interpreted correctly. The CT revealed typical features for PMP like ruptured and calcified appendix, massive mucinous ascites, small bowel narrowing and distortion, central displacement of the small bowel, high attenuation elements within the mucous, implied visceral scalloping of the liver, and to a lesser extent omental cake [10]. Tumor markers may be helpful in the work-up of PMP, the recommended are CEA, carbohydrate antigen 125 (CA125), and carbohydrate antigen 19.9 (CA19.9). Elevation in one or more of these predicts poorer disease-free survival (DFS) and overall survival (OS). Our patient had not all of these taken before the diagnosis was established, but completed later. Both CEA and CA19.9 was elevated, but not CA125 [11].

Modern treatment of PMP consists of CRS and HIPEC [12–14]. All macroscopically visible tumor masses within the peritoneal cavity are surgically removed if possible, and the patients receive subsequent perfusion of the abdominal cavity/peritoneum with heated chemotherapy, while still in general anesthesia [15]. Perioperative scoring of PCI is accepted as the best metric to quantify the extent of peritoneal disease [16], as CT scan has been shown to have a low sensitivity in detecting small peritoneal lesions [17]. The index’ range is from zero, which is no visible tumor manifestations, to 39, with tumor in all parts of the peritoneum. The presented patient had a PCI estimated to 28 of 39. Many centers, treating peritoneal metastasis (PM) from colorectal cancer (CRC), have an upper limit of PCI from 20 to 22/39 for CRS-HIPEC. A much higher PCI is in general accepted for PMP patients, as CRS-HIPEC is shown to be beneficial with higher PCI in PMP patients than in those with PM-CRC. PCI seems to be the most important predictor of survival in PM-CRC [18, 19], while PCI as a predictor in PMP is disputed. Some authors have not found correlation with survival even in peritoneal dissemination from high-grade appendiceal primaries [20], while others have found high PCI as a predictor for poorer progression-free survival (PFS) [21]. Thus, the presented patient had an acceptable PCI in respect to indication for CRS-HIPEC in PMP.

Completeness of cytoreduction (CC) is a term used in CRS and is assessed at the end of surgery by measuring the diameter of the largest remaining tumor deposits [22]. Complete removal of all visible tumor of the peritoneum is termed CC0, and CC1 refers to residual disease less than 0.25 cm. CC2 and CC3 refers to residues between 0.25–2.5 cm and > 2.5 cm, respectively. Both CC0 and CC1 are considered as complete cytoreductive surgery in PMP. The rational for HIPEC is targeting of residual microscopic and macroscopic disease ideally less than 0.2–0.3 cm in diameter. The presented patient had a CC from 1 to 2, as some residual mucinous deposits behind the liver in proximity to the piggyback anastomosis to the inferior vena cava, an area which is usually retroperitoneal, was left due to safety reasons.

Chronic immunosuppression has been associated with poorer prognosis in CRC patients undergoing surgery, in particular in respect to the risk of developing distant metastasis [23]. There is emerging evidence pointing to LTX as a safe treatment option and with a far better prognosis than chemotherapy in selected patients with non-respectable liver-only CRC-metastases [24]. In addition, growth rate of pulmonary CRC-metastases in LTX patients has been shown to be approximately the same as in a non-transplanted control group [25]. Thus, there are indications that immunosuppression not necessarily worsen the prognosis in CRC-metastatic patients. As PMP is a far less aggressive malignancy than CRC, major surgery with HIPEC should not be excluded as a treatment option in immunosuppressed patients even with an abdominal solid organ graft.

## Conclusion

To our knowledge, this is the first publication on CRS-HIPEC for PMP in an immunosuppressed liver-transplanted patient. One case report on CRS-HIPEC in a liver-transplanted patient with PM-CRC is previously published and concluded with feasibility of the procedure in this patient category [26]. The presented case shows that CRS-HIPEC after LTX is feasible in PMP with high PCI, without harm to the transplanted organ, with manageable postoperative complications, and adds to the knowledge of major abdominal surgery including HIPEC after LTX.

## Abbreviations

CA125: Carbohydrate antigen 125
CA19.9: Carbohydrate antigen 19.9
CC: Completeness of cytoreduction
CEA: Carcinoembryonic antigen
CRC: Colorectal cancer
CRS: Cytoreductive surgery
CT: Computed tomography
DFS: Disease-free survival
ERCP: Endoscopic retrograde cholangiopancreatography
HIPEC: Hyperthermic intraperitoneal chemotherapy
LTX: Liver transplantation
MRI: Magnetic resonance imaging
OS: Overall survival
PCI: Peritoneal cancer index
PM: Peritoneal metastasis
PMP: Pseudomyxoma peritonei
PSC: Primary sclerosing cholangitis
US: Ultrasound
